# The Socioecology of Territory Size and a "Work-Around" Hypothesis for the Adoption of Farming

**DOI:** 10.1371/journal.pone.0158743

**Published:** 2016-07-08

**Authors:** Jacob Freeman

**Affiliations:** Anthropology Program, Utah State University, Logan, UT 84322, United States of America; University College Dublin, IRELAND

## Abstract

This paper combines theory from ecology and anthropology to investigate variation in the territory sizes of subsistence oriented agricultural societies. The results indicate that population and the dependence of individuals within a society on “wild” foods partly determine the territory sizes of agricultural societies. In contrast, the productivity of an agroecosystem is not an important determinant of territory size. A comparison of the population-territory size scaling dynamics of agricultural societies and human foragers indicates that foragers and farmers face the same constraints on their ability to expand their territory and intensify their use of resources within a territory. However, the higher density of food in an agroecosystem allows farmers, on average, to live at much higher population densities than human foragers. These macroecological patterns are consistent with a “work-around hypothesis” for the adoption of farming. This hypothesis is that as residential groups of foragers increase in size, farming can sometimes better reduce the tension between an individual’s autonomy over resources and the need for social groups to function to provide public goods like defense and information.

## Introduction

The intensification of land use is a process associated with the evolution of human societies (e.g., [1–9]). The intensity of human land use is a consequence of how social groups organize to harvest and distribute resources. Although ecologists have long studied the home-ranges of animals to investigate the processes that cause and constrain how animals harvest and distribute resources (e.g., [10–15]), there has traditionally been very little cross-fertilization between anthropological studies of intensification and ecological studies of animal home-ranges. However, this is starting to change. Two recent papers have adopted a model of energy flux drawn from animal ecology to explain the sizes of hunter gatherer ethnic territories (group home-ranges) [16, 17], and these studies provide a new way to view the dynamics of hunter-gatherer land use. The pertinent observation here is that the size of group home-ranges among ethnographically documented societies is, under the right technological circumstances, a sub-linear function of population size [16, 17] (see [16, 18] for possible explanations).

The sub-linear scaling of ethno-linguistic population and territory size simply means that a one unit increase in population size results in an increase in territory size that is less than one unit, and the marginal increase in territory size declines as populations become larger. For example, let’s say territory size increases three fourths as fast as population size. Then, if a population of 10 individuals using 5.6 *km*^2^ doubles to a population of 20, territory size will only increase to 9.4 *km*^2^. The consequences of this relationship are important, and, perhaps, under-appreciated. The sub-linear scaling of ethno-linguistic population and territory size indicates that as ethnic populations grow, local residential groups fission at a higher group size threshold and, thus, become larger [16]. In conjunction with increases in group size, as hunter-gatherer ethno-linguistic populations increase, rather than simply spreading out in space to find more food, residential groups harvest food from the territory they already use more intensely and effectively.

Living in larger residential groups that extract more resources more effectively from local ecosystems, however, is not a panacea for coping with population growth at larger scales of space and time. Larger residential groups accommodate population growth, but this also means more conflicts of interest among individual foragers over access to resources and mates, resource depression that can lead to threshold changes in ecosystems [1], and more costly communication [19, 20]. It is these tradeoffs that underlie a “work-around hypothesis” for specialization in the cultivation of domesticated plants at the expense of hunting and gathering.

In anthropology, the work-around hypothesis was originally proposed by Richerson and Boyd [21] to explain the evolution of large, complex societies. Their thesis is succinct: Cultural group selection, under conditions of increasing population, favors institutions that make living in large-scale groups possible while preserving a sense that individuals live in small-scale, egalitarian societies [21]. In particular, institutions are favored that minimize an underlying tension between an individual maintaining a social psychology of autonomy and egalitarian relationships and the ability of a group of unrelated individuals to produce public goods (e.g., defense, roads, & sanitation) that are critical to the maintenance of large, state societies. My thesis here is a simple extension of the work-around hypothesis to smaller-scale societies and the decision to farm: Under some social-ecological conditions, foragers begin to specialize in farming to minimize the tension of living in larger residential groups caused by increases in the size of an ethno-linguistic population. More generally, any selective pressure that favors increases in residential group size will also favor the adoption of strategies, whether they be new institutions or technologies, that minimize the tension between an individual’s autonomy over resources and living in residential groups that function, at least sometimes, to provide collective benefits (e.g., defense or information).

This hypothesis suggests a simple prediction that is testable with macroecological data on human societies: Foragers and farmers face the same constraints on their ability to harvest and distribute resources. This is to say that, under conditions of population growth, farmers are no more efficient than foragers at extracting resources per person from a territory. Thus, the scaling relationship between population and territory size should be equivalent, regardless of subsistence type. However, an increased investment in farming increases the energy density of an ecosystem, which allows individual farmers to use smaller home-ranges than individual foragers *at a given group size*. In short, the use of less space per person, at a given group size, allows individual farmers to retain more autonomy over their production of food and reduces the tension between an individual’s desire to maintain autonomy and the ability of social groups to function.

To explore the plausibility of the work-around hypothesis for increased investment in farming, in the rest of this paper I answer two questions. (1) Consistent with human foragers, do population size, diet and the productivity of resources determine the territory sizes of ethno-linguistic populations of farmers? If yes, in particular with respect to population, then (2) is the scaling relationship between territory size and population less than or the same as the scaling observed in hunter-gatherer societies? The answers to these questions suggest that foragers and farmers face similar constraints on their use of space and similar incentives to live in larger residential groups, rather than simply fission and spread-out in space, as the size of an ethno-linguistic population increases. This is consistent with the work-around hypothesis. The role of conflicts of interest generated by increases in residential group size is an under-appreciated mechanism that may sometimes favor a shift from foraging to farming based economies.

### A Model of Territory Size in Agricultural Societies

Following models of hunter-gatherer territory size [16, 17], I use a production model to explain the territory sizes of subsistence level agricultural societies. I assume that the territorial extent of an agricultural society is a function of the area necessary for an individual to produce food multiplied by the population of a social group:

A=a(P,Q)N. (1)

Here, *A* is the territory (*km*^2^) of a social group; the function *a*(*P*, *Q*) defines the amount of space that an individual needs (*km*^2^) to satisfy her demand for food. *P* is the productivity of an agroecological system, and *Q* defines how effectively individuals find food on a landscape, which is determined by their technological capacity and diet. Finally, *N* is the population size of an ethno-linguistic group.

For simplicity, I assume that *a* is a function of resource supply and demand. Thus,

$$a\left(P,Q\right)=\frac{E_{d}}{E_{s}}Q$$ (2)

where *E*_*d*_ is an individual’s demand for energy (e.g., Kcals/day), and *E*_*s*_ is the energy production density (e.g., Kcals/*km*^2^) of an agroecological system. Following Freeman and Anderies [16], I treat *E*_*d*_ as constant for a given population. Future models can improve this assumption by taking into account variation in body size and the energy required for individuals to perform rituals and ceremonies. However, for the baseline analysis conducted here, the assumption is reasonable (see below).

The density of energy in an agroecosystem depends, in part, on the availability of solar energy and water for plant growth. Actual evapotranspiration is a simultaneous measure of energy and water availability and defines the balance of water in an agroecosystem. I assume here that for a constant level of time investment in the production of crops by an individual, the energy density of an agroecosystem is an increasing, diminishing returns function of actual evapotranspiration. This is formalized as $P=C_{1}P^{\beta_{1}}$. Where *C*_1_ is a constant, and *β*_1_ is an exponent that defines the change in energy density per unit change in *P*. This relationship between *P* and crop yield is highly general and has empirical support [22]. The above assumptions help operationalize the energetic dynamics of agricultural societies consistent with the underlying energetic model of territory size in hunter-gatherer societies.

Not every society that practices agriculture is equally specialized in the production of domesticated plants. Members of agricultural societies may also forage for wild foods, raise live stock and/or purchase food. Given the sample analyzed here; foraging is the most relevant alternative subsistence pursuit to consider [23].

I assume that the productivity of agricultural fields in an agroecosystem is inversely related to the amount of time that individual farmers spend on alternative subsistence activities [e.g., 24–27]. For example, the more time that individuals spend foraging for wild foods, the less time they can devote to weeding, guarding, watering and harvesting crops, and this reduces the productivity of a given field. Thus, there is a time allocation trade-off between foraging for food and the productivity of fields/gardens. The more time that individuals spend foraging, the greater the amount of territory they should need to find food. This should occur for two reasons: (1) The productivity of fields/gardens declines and (2) individuals need to cover more area to effectively locate wild food. I, thus, define the rate of energy production in an environment as the supply of energy, *E*_*s*_ times how effectively individuals use their time to locate and produce food, *Q*. The more that individuals forage for food, the less effectively they produce crops for food because they must search for and travel to wild foods that are in dispersed locations. The effectiveness of food acquisition is defined here as $Q=c_{2}e^{-\beta_{2}F}$, where *c*_2_ is a constant, *e* is the exponential, 0 < *β*_2_ < 1, and *F* is the contribution of foraging to an individual’s subsistence. This expression assumes that as individuals forage more, they get exponentially less effective at producing crops because they must use their time to search for and travel to find “wild” resources.

Given the above assumptions, the space needed by an individual farmer can be written as:

$$a\left(P,Q\right)=\frac{E_{d}}{c_{1}c_{2}P^{\beta_{1}}e^{-\beta_{2}F}}.$$ (3)

Further, given that an ethno-linguistic group’s territory depends only on each individual’s use of space to produce food, then Eq (3) can be used to define a group’s territory as

$$A=a\left(P,Q\right)N=\left(\frac{E_{d}}{c_{1}c_{2}}\right)P^{-\beta_{1}}e^{\beta_{2}F}N.$$ (4)

One can simplify Eq 4 by letting *C* = *E*_*d*_/*c*_1_ *c*_2_, and, hence, the territory of an ethno-linguistic group can be written as:

$$A=CP^{-\beta_{1}}e^{\beta_{2}F}N.$$ (5)

As described by others [16, 18], Eq (5) implicitly assumes that the use of space by individuals is independent. If this is true, then territory will scale linearly with population. However, we know that in social groups individuals are not independent, autonomous actors in their quest for food, and previous work indicates that the scaling relationship between population and territory size might be sub-linear in agricultural societies [28]. A simple way to account for this possibility is to write $N^{\beta_{3}}$, where *β*_3_ is an exponent that scales the relationship between population and territory size. If the relationship is linear, then *β*_3_ = 1. However, the relationship could be super-linear (*β*_3_ > 1) if, for example, individuals fight over the placement of their fields and, as a result, spread out in space to avoid conflicts over field locations. Finally, the relationship could be sub-linear (*β*_3_ < 1) if, for example, individuals cooperate to guard and invest more time in tending fields, and the result of such activities is that fields become more productive per person per unit of territory. Hence, Eq (5) becomes

$$A=CP^{-\beta_{1}}e^{\beta_{3}F}N^{\beta_{3}}.$$ (6)

### Predictions

Given that the same production function described by Eq 6 also describes the territory size of hunter-gatherers [16], I make two sets of predictions. The first set (1a–1c) is specific to agricultural societies and is captured by Eq 6:

1a. Productivity has a negative effect on territory size.
1b. The percent of diet from foraging has a positive effect on territory size.
1c. Population size has a positive effect on territory size.

If the data are consistent with at least one of these predictions, it would indicate that the model of energy flux used to describe animal and hunter-gatherer home-range size is also, at least partly, useful for explaining the group home-ranges of agricultural societies.

The second set of predictions (2a & 2b below) relates to the scaling of population and territory size in forager vs. farmer societies, and the values of the constant, *C* in Eq 6 in forager vs. farmer societies. The basic premise of the work-around hypothesis is that farmers are not better at harvesting energy and information from a territory than foragers. Rather, any increase in population size at the level of the ethno-linguistic group will put pressure on individuals, whether foragers or farmers, to live in larger residential groups and harvest resources more intensely from local ecosystems. This process generates economies of scale.

However, larger residential groups of foragers create more conflicts of interest for individuals, raising the tension between what is best for individuals, in terms of their autonomy over production, and the ability of local groups to function (i.e., work in concert). As Richerson and Boyd [21] describe the tension:

“The tension between the small-scale loyalties dictated by self-interest, kin selection, and reciprocity, and the larger-scale loyalties generated by tribal institutions, is unresolved in humans…Thus, humans are adapted to tolerate a system in which there is conflict among the cooperators, as evidenced by such behavior as the patient search for consensus in forager communities (and university committees). Institutions that minimize the conflict inherent in the gene-culture system will be favored by the processes of cultural evolution, but these institutions cannot, in the nature of the situation, be perfect”

(p. 258).

Population growth in hunter-gatherer ethno-linguistic groups increases this tension between self-interest and cooperation because residential groups of foragers become larger and extract resources more intensely. Hence, I propose that an investment in farming, at the expense of foraging, can best reduce the tension, allowing individuals to maintain autonomy over production and cooperate in activities like defense, information exchange and mate exchange.

My hypothesis has two implications. The first is that farming does not have an advantage over foraging for individuals in terms of how efficiently an individual produces food (Kcals/time). Rather, the advantage of farming, under some circumstances, is that it increases the density of resources in an ecosystem and reduces conflicts of interest over resources at a given group size, giving individuals more personal control over production. This may fly in the face of the established anthropological paradigm that the adoption of agriculture changes the dynamics of human-environment interactions, but this premise is consistent with the energetic theory of home-range developed here and elsewhere [16, 17, 29]. As Holling [14] succinctly predicts,

“mammals of the same body mass, with the same longevity and metabolic rates and efficiencies, will have foraging areas with a spatial extent determined by the productivity of the landscape and by their trophic status. The less productive the landscape and the farther up the trophic chain, the larger the spatial extent of their foraging areas”

(p. 472).

In other words, given that foragers and farmers have very similar body masses and metabolic rates, foragers and farmers should face the same energetic constraints on their ability to harvest and distribute resources within a territory as populations increase in size. This does not deny that body mass varies among ethnographiclly recoded societies. I am only assuming that the variation in body mass is very small relative to variation in the energy density of the ecosystems that foragers and farmers create and variation in population size. Thus, I predict:

2a. The scaling coefficient that relates population and territory size should be the same when forager and farmer societies are compared.
2b. However, the production of crops increases the potential energy density of an ecosystem, thus the area needed per person, holding population constant, should be lower in farmer vs. forager societies.

If the data are consistent with these two predictions, it would indicate that the work-around hypothesis is consistent with basic macroecological patterns in human ecology and is plausible. However, if the population-territory scaling coefficient among farmers is less than the coefficient observed among human foragers, then this would indicate that farming has an inherent advantage, in terms of productivity per person per unit of territory, over foraging under conditions of population growth. Such a result would contradict the work-around hypothesis for the adoption of farming.

## Materials and Methods

A sample of 61 ethnographically recorded small-scale agricultural societies is analyzed here. Data on 50 of these groups were originally collected to study the determinants of crop richness in small-scale agricultural societies [23]. I augmented this sample by collecting data on 11 additional small-scale farming societies listed in the Ethnographic Atlas [30]. The sample represents a wide range of known latitudes at which subsistence farmers are recorded in the ethnographic record, and, though the sample is not exhaustive, the sample size is sufficient to begin to describe large-scale patterns.

Estimates of population (N) and territory size (A) occupied by agricultural societies were collected from primary ethnographic sources. The population values for each case were reported by each ethnographer either as a result of their own census efforts or a population census undertaken by national governments. The territory occupied by each social group was estimated by each ethnographer or from territorial maps provided by each ethnographer, and all area values were converted to square kilometers. The population and area estimates given by each ethnographer were made independently in different times and places.

I use the proportion of a normalized individual’s diet from foraging (hunting, gathering wild plants and fishing) to estimate commitment to foraging. The greater the proportion of diet from foraging, I assume, the less time a normalized individual invests in producing crops [23].

I estimate the productivity of agroecosystems using actual evapotranspiration, which is a simultaneous measure of solar energy and the availability of water. Estimates for annual actual evapotranspiration were obtained from the Food and Agriculture Organization for the globe at a five arch minute scale [31]. Territories estimated as circular polygons for each society were then layered over global actual evapotranspiration data and the value closest to the center of a group’s range was recorded. All of the data and ethnographic references are available in the supplemental files.

To determine the effects of productivity, foraging and population on territory size among agricultural societies, I use a linear model. That is, I transform Eq 6 by taking the log of both the right and left hand sides. This gives the equation

$$\ln\;A=\ln\;C-\beta_{1}\;\left(\ln\;P\right)+\beta_{2}F+\beta_{3}\;\left(\ln N\right)+e$$ (7)

where *A* is the territory size of a given society; *β*_1…3_ is the coefficient associated with each respective explanatory variable; and *e* is the residual error not explained by the three explanatory variables.

I use model selection methods to evaluate the value, sign and relative importance of the coefficients associated with each explanatory variable [32]. I base my model selection analysis on the Akaike Information Criterion (AIC), which is a measure of the fit and complexity of a statistical model. I use the MuMin package in R to estimate all potential regression models for the set of explanatory variables identified in Eq 7. Each model is ranked according to its AIC value from lowest to highest AIC. The best model is the regression model with the lowest AIC. This ranking is used to calculate the change in AIC, Δ_*AIC*_, as *AIC*_*i*_ − *minAIC*, where *AIC*_*i*_ is the AIC of a candidate model under consideration and *minAIC* is the AIC of the model that best balances fit and complexity.

I use standardized Akaike weights, *w*_*i*_ calculated for each regression model to summarize the likelihood that a given model is the best approximate fit, given the data and other candidate regression models. The Akaike weight is calculated by first determining the likelihood that a model is the best approximation to the data, which is given by: $L(model|data)\;\propto \;e^{0.5\Delta_{i}}$. Next, the the sum of the likelihoods of all regression models is calculated. Then, the Akaike weight is simply $w_{i}=\frac{e^{0.5\Delta_{i}}}{\sum_{r=1}^{R}e^{0.5\Delta_{r}}}$. I use the Akaike weight to define a 99% confidence set of models. This is the set of models that has a 99% chance of containing the regression model that best fits the data.

To evaluate the likelihood that productivity, diet and population have an effect on territory size, I calculate the relative importance of each variable present in the 99% confidence set of regression models. The relative importance of an explanatory variable is the sum of the Akaike weights of each model in which a variable is present. To illustrate, if the 99% confidence set of regression models contains three candidate models, each model with a weight of 0.80, 0.10 and 0.10, and population is an explanatory variable in all three models while actual evapotranspiration is only an explanatory variable in the top two, then population’s relative importance would equal 1 and actual evapotranspiration’s would equal 0.90. In this case, population would be 1.11 times more likely to have an effect on territory size than actual evapotranspiration (1/0.90). The closer a variable’s importance measure is to 1, the more likely the variable is to have a true effect on the response variable, given the data and candidate set of regression models.

To compare foragers and farmers, I use the same regression and model selection procedure outlined above to estimate the average scaling coefficient for population and territory size and the average intercept of 339 hunter-gather societies [2]. Specifically, I start with the production model developed for hunter-gatherers by Freeman and Anderies [16],

$$A=Cr^{-\beta_{1}}e^{-\beta_{2}T}e^{\beta_{3}H}N^{\beta_{4}}$$ (8)

where *C* is a constant; e is the exponential; r is rainfall; T is temperature; H is the proportion of diet from hunting; and N is population size. Note that *r* and *T*, together, estimate the productivity of wild foods. *H* controls for diet (trophic level), and N is population size. Taking the log of the right and left hand sides of the above equation gives a linear production model of territory size for hunter-gatherers that is analogous to Eq 7.

$$\ln\;A=\ln\;C-\beta_{1}\;\left(\ln\;r\right)-\beta_{2}T+\beta_{3}H+\beta_{4}\;\left(\ln N\right)+e$$ (9)

where *A* is territory size; *β*_1…4_ is the coefficient associated with each respective explanatory variable; and e is the residual error.

I calculate a Z-score to determine the likelihood that the scaling coefficient of population and territory size in agricultural and hunter-gatherer societies are equal [33]. Succinctly, the Z-score is:

$$Z=\frac{\beta^{i}-\beta^{j}}{\sqrt{SE_{i}^{2}+SE_{j}^{2}}}$$ (10)

where *β*^*i*^ and *β*^*j*^ are the population-territory size scaling coefficients of agriculturalists and hunter-gatherers, respectively. *SE*_*i*_ and *SE*_*j*_ are the standard errors associated with each respective coefficient. The Z-score is useful to evaluate the null hypothesis that the two scaling coefficients (*β*_3_ in Eq 7 and *β*_4_ in Eq 9), drawn from different populations, are the same. To reduce the risk of type II error, I set *α* = 0.1 meaning that a p-value <0.1 is sufficient to reject the null-hypothesis. I also use a Z-score to evaluate the null hypothesis that the intercepts, *C* in Eqs 7 and 9, are equal.

Finally, a study of spatial autocorrelation in the best regression models identified during model selection revealed very weak spatial autocorrelation. Spatial autocorrelation could bias coefficient estimates upward. Recall, one of my goals is to compare the population-territory size coefficients of hunter-gatherer and agricultural societies. If the coefficient that relates population and territory size were biased upward by autocorrelation, this would taint the comparison. I found, however, that this was not a significant concern because population coefficients did not change in spatially lagged regression models (see S1 Text). All of the data used in this study are available in the Supporting Information (S1 and S2 Tables) for the purpose of study replication and reanalysis.

## Results

My analysis illustrates four results that are consistent, or at least not inconsistent with, the work-around hypothesis. I first summarize the results and then detail the evidence that supports these results below.

1. Population (*N*) and the percent of diet obtained from foraging (*F*) partly determine the territory size of agricultural societies.
2. Conversely, the productivity of an agroecosystem (estimated by actual evapotransipriation) is unlikely, at a global scale, to determine the territory sizes of subsistence oriented agricultural societies.
3. The scaling-relationship between population and territory size is sub-linear among agricultural societies. This suggests that as the population of ethno-linguistic groups increases, farmers live in larger residential groups and produce more food per unit area per person.
4. The scaling relationship between population and territory size is likely the same in small-scale agricultural societies and hunter-gatherer societies, but, holding population constant, farmers use less area than foragers. This means that foragers and farmers face the same constraints on their ability to harvest more resources per person from a territory. Farmers simply have a higher potential carrying capacity than foragers.

### Results one through three: The determinants of territory size in agricultural societies

In general, we can have a very high degree of confidence that population and the percent of diet obtained from foraging affect the territory size of agricultural societies (result one above). However, it is highly uncertain whether the productivity of an agroecosystem affects territory size, at least at a global scale (result two above). These results are illustrated by Tables 1 and 2.

**Table 1:** A summary of model statistics for the 99% confidence set of regression models. F = the percent of diet from wild foods; N = population, P = actual evapotranspiration.

Model #	Variables	*df*	*AIC*	Δ_*AIC*_	*W*_*i*_	*R*^2^
1	*F*, *N*	4	243.0	-	0.729	0.66
2	*F*, *N*, *P*	5	245.0	2.0	0.270	0.66
3	*N*	3	277.1	34.1	0.001	0.38

**Table 2:** Mean coefficients, standard errors and relative importance (∑*w*_*i*_ for each variable) of the explanatory variables in the 99% confidence set of models (n = 61).

Explanatory Variable	Coefficient (*b*)	Standard Error	*Importance*
*Intercept*	-1.31	2.22	–
Population (*N*)	0.81	0.09	1.00
Foraging (*F*)	0.07	0.01	1.00
Productivity (*P*)	-0.07	0.55	0.27

Table 1 presents the 99% confidence set of regression models that best fit the data on agricultural societies. Model #1 includes population and the percent of diet obtained from foraging. This model explains 66% of the variation in territory size and is approximately 2.7 times more likely (0.721/0.270) to best fit the data than model #2. An important observation is that population is an explanatory variable in all three of the regression models in the 99% confidence set. This illustrates the importance of population size for explaining the territorial extents of subsistence agriculturalists. The percent of diet obtained from foraging is also an explanatory variable in the top two models in the 99% confidence set, illustrating that this variable is also quite important in this data set.

Table 2 illustrates the model averaged coefficients, standard errors and the summed Akaike weights (importance) of each of the explanatory variables in the 99% confidence set of regression models. Consistent with the model of territory size outlined by Eq 6, with a coefficient of 0.81 population has a positive effect on territory size (prediction 1c), and with a coefficient of 0.07, the percent of diet obtained from foraging has a positive effect on territory size (prediction 1b). The direction of the effect of productivity on territory size is negative, which is consistent with prediction 1a, but the negative effect is highly uncertain. For example, population and the percent of diet from foraging are 3.7 times (1/0.27) more likely than actual evapotranspiration to have an effect on territory size, in this data set. This suggests that, at a global scale, actual evapotranspiration is unlikely to partly determine the spatial extents of agricultural societies.

**Fig 1:**
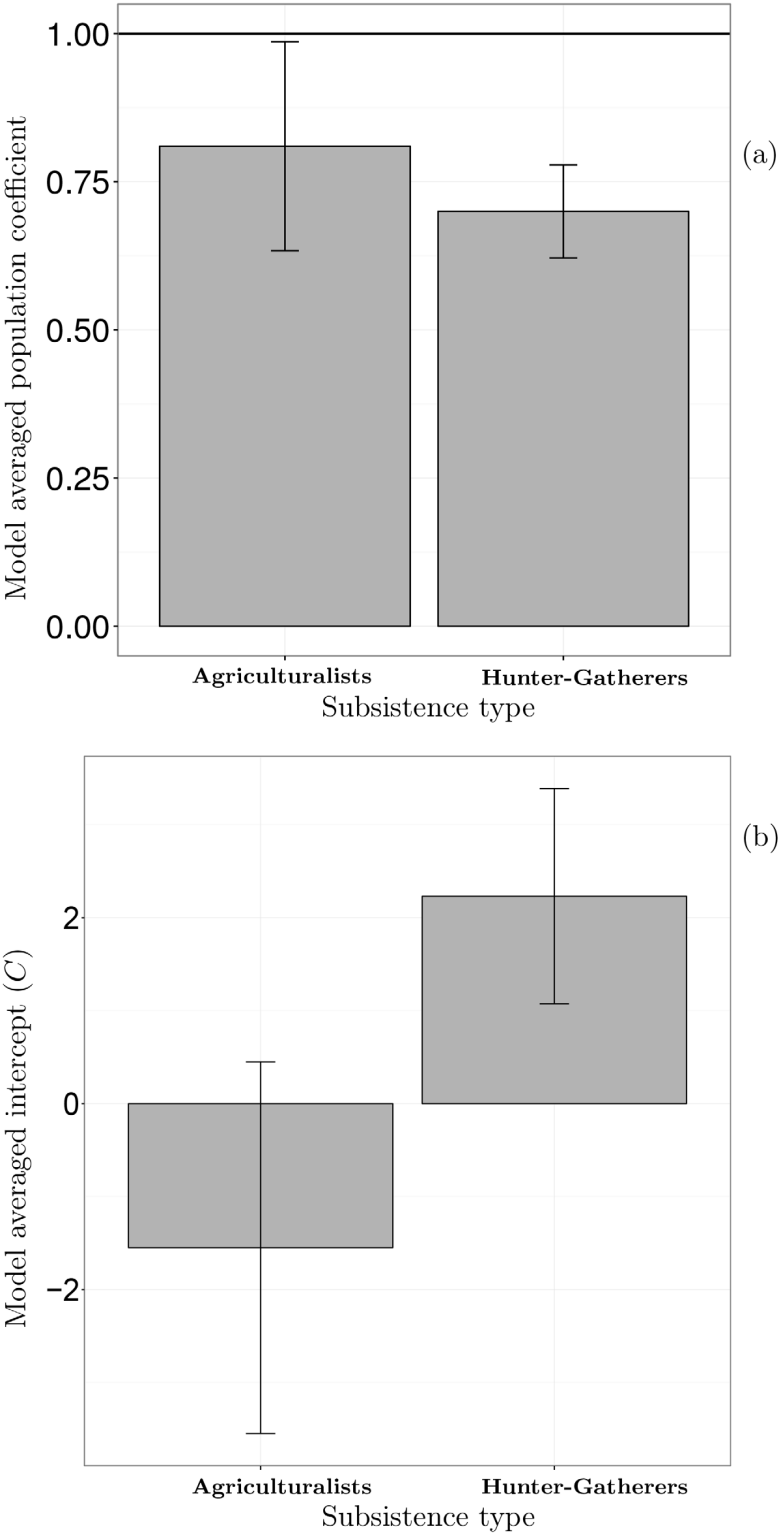
Model averaged population coefficients and intercepts from regression Eqs 7 and 9 and the 95% confidence interval for each coefficient or intercept shown as error bars for each subsistence type.

Finally, the model averaged coefficient associated with population is 0.81, and the standard error is 0.09. Thus, the 95% confidence interval for the population coefficient is 0.64-to-0.98 (Fig 1a). This indicates that the coefficient is likely less than one. Thus, we might infer that as ethno-linguistic groups of farmers increase in size, farmers harvest more resources per person from a territory, and, consequently, farmers use less space to produce food as ethno-linguistic populations increase in size, but residential groups within ethnic populations likely also become larger.

### Result four: A comparison of foragers and farmers

Fig 1 illustrates evidence that the population-territory size scaling coefficients among forager and farmer societies are likely the same, or at least we cannot reject this possibility. However, the intercepts, *C* in Eqs 7 and 9, are different, with *C*_*farmers*_ < *C*_*forgers*_. These results are consistent with predictions 2a and 2b, and suggest that foragers and farmers face the same constraints on their ability to harvest resources from a territory as populations increase in size. However, holding population size constant, farmers use less space per person and, thus, social groups of farmers have smaller territories than social groups of foragers who live at the same population.

As Fig 1a illustrates, the mean population-territory size scaling coefficient among agricultural societies is 0.81 (*S*.*E*. = 0.09), and the coefficient among foragers is 0.70 (*S*.*E*. = 0.04). A Z-score of 1.12 indicates that there is a 13% chance that these coefficients are the same. In this case, because *p* = 0.13 > *α* = 0.1, there is insufficient evidence to reject the null hypothesis that these two coefficients are the same. Conversely, Fig 1b illustrates that the model averaged intercepts are quite different. The intercept among farmers is -1.31 (*S*.*E*. = 2.22) and the intercept among foragers is 2.23 (*S*.*E*. = 0.59). With a Z-score of 1.6, there is only a 0.054% chance that these two intercepts are the same value. In this case, because *p* = 0.05 < *α* = 0.1, there is sufficient evidence to reject the null hypothesis that these two intercepts are the same.

**Fig 2:**
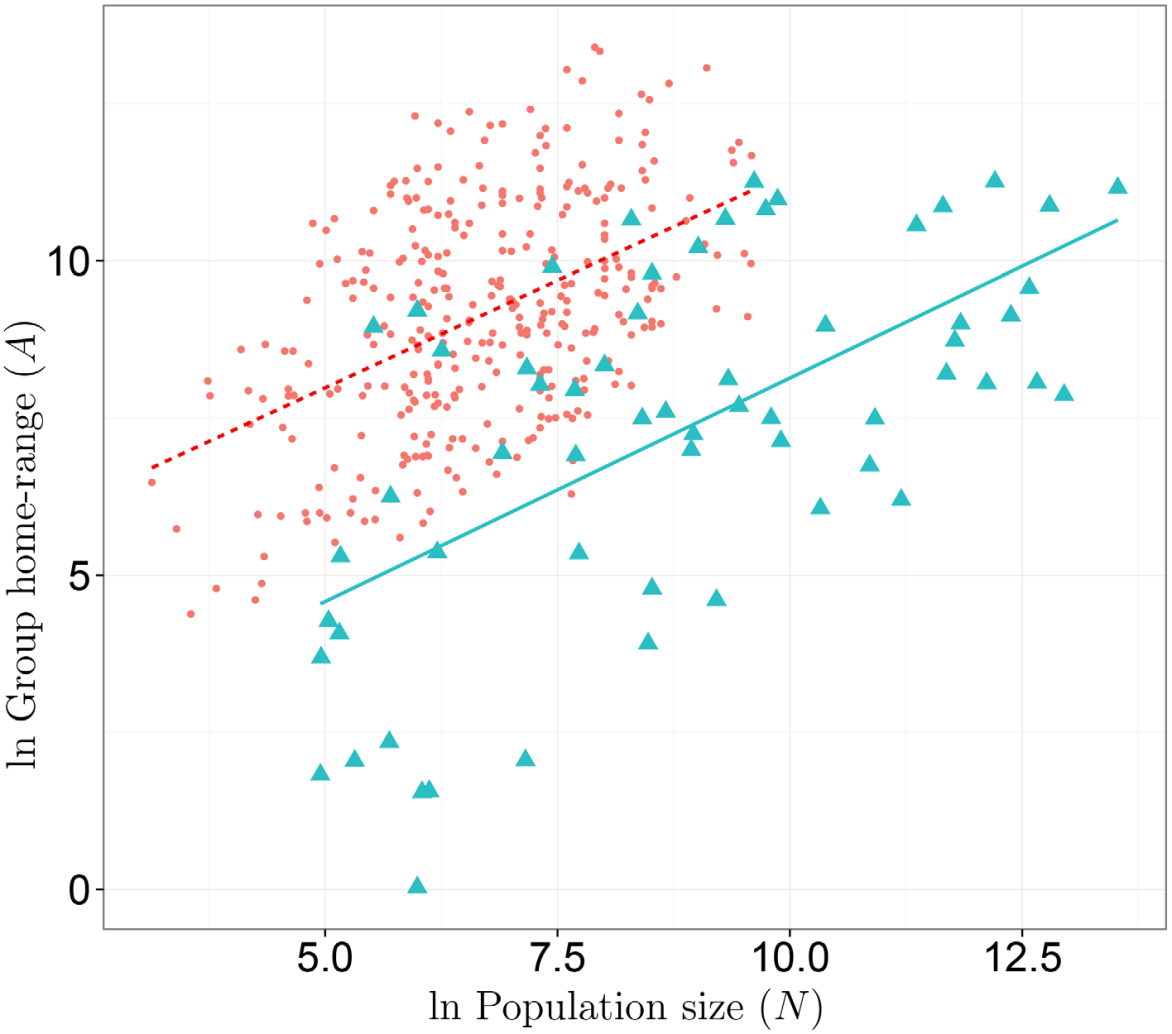
A comparison of the group home-range and population size regression lines for hunter-gatherer and agricultural societies. dots = hunter-gatherer societies, and triangles = agricultural societies. The dashed line is an OLS regression line for hunter-gatherers, the solid line is the same for agriculturalists.

Fig 2 provides a way to visualize the results presented in Fig 1. Just by visual inspection, one can observe that the slope of the line that relates population and territory size is the same among foragers (dashed line and dots) and farmers (solid line and triangles). However, on average, among equivalent sizes of ethno-linguistic populations of foragers and farmers, the farmers live in smaller territories than foragers. Thus, the solid line will meet the y-axis at a lower value than the dashed line meets the y-axis. Clearly, and most interestingly from the standpoint of the work-around hypothesis, there is also overlap in terms of population size and territory size (i.e., foragers and farmers living at the same population densities). As I discuss below, this is consistent with the premise of the work-around hypothesis that farming is a contextual adaption made in some ecosystems by local residential groups. It is a local response to the general (at least among small-scale foragers and farmers) way that residential groups increase in size and individuals produce more resources per person when they experience population growth at larger scales. In some environments, under conditions of ethno-linguistic population growth, farming best reduces the tension between individual autonomy and group function, and, in others, strategies historically labeled as foraging best reduce this tension.

## Discussion

The recent application of models of energy flux drawn from animal ecology helps explain the territory sizes of hunter-gatherers and provides a new way of observing the process of intensification in human societies [16–18]. An important observation is that territory size is a sub-linear function of population among forager societies. This indicates that foragers respond to increases in population at large scales by living in larger residential groups at more local scales and investing in technologies and networks that allow individuals to more intensely harvest resources per person from a territory. This macroecological pattern, thus, reflects one way that populations of foragers adapt to population growth. However, while living in larger residential groups that more intensively use a territory is one way that foragers can respond to population growth at large scales, more local-scale residential groups should experience an increased tension between individual self-interest and the ability of social groups to function as a whole.

I propose that cultural evolution may favor more investment in farming at the expense of hunting and gathering to work-around this increasing tension in the spirit of Richerson and Boyd’s original work-around hypothesis for the evolution of states [21]. A simple way to think about this argument is that once residential groups of foragers become large enough, the adoption of farming gives individuals more autonomy over their production of food and the ability to enforce egalitarian social relationships, even while larger groups composed of less related individuals cooperate for purposes such as defense, information and mate exchange.

For example, consider the well known social logic of mobile hunter-gatherer societies. There is always a tension between two competing principles: precedent and commitment to the group. The logic of precedent simply means that an individual or some individuals have first right to a resource, and, in many societies, it is this principle that becomes the foundation of debt and structural inequality [34]. We see this principle of precedent in hunter-gatherer societies in ethnographic statements like: “Among Western Shoshoni the household was very nearly a self-sufficient economic unit. With the exception of large game, all foods belonged to the households of the persons acquiring them” [35](p. 239). Similarly, in the Kalahari, Marshall states that “Once the Veldkos is gathered it belongs to the person who gathered it” [36](p. 335). The same principle of precedent applies to territories, with custodians of a territory, and thus those with rights to harvest resources, acknowledged based on established residence [36]. The principle of precedent, however, is balanced by sharing, which, at least tacitly, recognizes the important benefits, such as information and mate exchange, necessarily provided by social groups.

When the two principles of precedence and commitment to the group (a kind of communism), which are always in tension, begin to cause too much conflict, it is the autonomy of individuals in mobile foraging societies over their production, and, thus, the ability to vote with their feet that reduces the tension. As Lee [37] describes,

“The camp is regarded by the Bushmen as a unit within which food is shared, and when sharing breaks down, it ceases to be a camp. Persons leave and others come in until the routines of subsistence are reestablished. Absolute sharing is the ideal of camp life but is rarely attained in practice. Fission and recombination of camps are the means by which the Bushmen can combine their search for food with a search for congenial camp mates”

(p. 350).

The point I am making is that because hunter-gatherers respond to population growth by living in larger residential groups that harvest resources more intensely (as evidenced by the sub-linear scaling of population and territory size), in some ecosystems it will be difficult for many individuals to maintain autonomy over their own production. Thus, it will be more difficult to balance the tension between individual interest and the need for a group to function because the logic of precedent may begin to overshadow commitment to the group. For example, as Lee [37] notes, camps that break apart are those in which sharing breaks down due to conflict. It does not take much to imagine how an increase in conflict would cause camp groups to coalesce and break apart much more frequently, resulting in a more rapid cycling of people through residential groups and territories on a landscape. Pretty soon, social discord will reign, and there will not be a suitable camp for many people, as disaffected individuals proliferate, causing cooperation to break down. The work-around hypothesis is that group selection will favor technologies or institutions in this situation that reduce social discord and allow groups to better function.

Farming can mitigate social tension brought on by the increasing incongruence between the logic of precedence and sharing demands, at least under some conditions, because it increases the energy density of a landscape. For example, the Tiv of Central Africa (in their core territory) live at a population density about 18 times higher than any ethnographically known hunter-gatherers. Yet, the Tiv maintain a flexible social organization in which individuals can move from minimal tar (the basic residential unit) to minimal tar. Their rights to land are established by residence, and any attempt to curtail such rights by ambitious men based on the logic of precedence is met by individuals voting with their feet. As Bohannan and Bohannan put it [38],

“The fear of “sitting alone” is acute among the Tiv. A man who wrangles with his sons and brothers soon finds himself ‘sitting alone,’ without labor, unable to work his fields. A bad temper or a surly, stingy nature ‘spoils the compound”’

(p. 83).

Tiv households have the right to sufficient land for production and establish this right through residence in a compound, and, thus, are constantly shifting compounds until they find a “good” compound head [38]. As with the mobile hunter-gatherers above, it is the ability of individual households to maintain autonomy over production that allows them to blunt the ability of individuals to establish too much in the way of prerogatives based on their precedent over resources.

To explore the plausibility, from an ecological standpoint, of the work-around hypothesis for a shift from foraging to farming based economies I asked two questions. First, do the same factors that determine territory size among hunter-gatherers and mammals in general also determine the territory size of subsistence-level agriculturalists? Consistent with human foragers, the population size of a social group and the composition of the diet of individuals within a social group both partly determine the territory sizes of agricultural societies. Together, population size and the percent of diet obtained from foraging for wild foods explain 66% of the variation in the territory sizes of agricultural societies (Tables 1 & 2). However, the annual balance of energy and water within an agroecosystem does not have a significant effect on the territory sizes of agricultural societies. This last result might be due to the fact that actual evapotransipriation is measured at too coarse of a grain in this study. More work is needed to determine the effects of agricultural productivity, as well as, additional factors on territory size in agricultural societies. Additional factors may include the availability of fuel, water and topographic heterogeneity. When fuel and water are scarce, territory size should decrease because individuals tether to places on a landscape where these scare resources are available. Increases in topographic gradients should increase the travel costs for individuals to move between resource patches and, thus, have a negative effect on territory size.

Second, given that population size and diet determine the territory sizes of agriculturalists, is the scaling of population and territory size among these two subsistence types equivalent? A basic assumption of the work-around hypothesis (for farming) is that subsistence-level (non-mechanized) farming does not have a per capita productivity advantage per unit of territory over foraging under conditions of population growth. If this assumption has an empirical basis, I reasoned that foragers and farmers should display an equivalent scaling coefficient between population and territory size. And my analysis supports this assumption (Fig 1a). Similarly, a key premise of my version of the work-around hypothesis is that, holding population size equal, farmers use less space per person than foragers because farming increases the energy density of an ecosystem, and this gives individuals a greater degree of autonomy over their production at a given population size. If this premise has an empirical basis, I reasoned that the energy density of the ecosystems that farmers create is, on average, higher than the energy density of the ecosystems that foragers create, and, thus, farmers use less territory per person than foragers. My analysis is consistent with this reasoning (Figs 1b & 2).

### Archaeological implications

The implication of the work-around hypothesis is that more commitment to the production of domesticated plants is a contextual rather than a proficient adaptation. This simply means that farming does not have a productivity advantage (Kcals/time/unit of territory) over foraging. Rather, in particular local contexts, specialization toward the production of domesticates has an advantage because farming better solves the tension between individual autonomy over resources and the ability of a social group to cooperate than alternative strategies. Where activities that anthropologists traditionally term hunting and gathering better solve this tension, we should observe hunter-gatherer adaptations in response to population growth. In short, I propose that population growth, or any process, like increases in the frequency of raids, that favors larger residential groups puts selective pressure on groups that favors institutions or technologies that best reduce the tension between what is best for individuals and what is best of the group.

From a temporal perspective, I imagine the following process: Mobile foragers experience population growth and begin to live in larger groups that produce food more effectively per person per unit of territory via economies of scale. Economies of scale increase the availability of energy in a society, but are not a panacea. Costs include increasing conflicts of interest over the distribution of resources, the potential for depletion to cause social-ecological systems to cross critical thresholds and increased competition for favored mates. These costs increase the tension between what is best for individuals and what is best for the group, mitigating the benefits of the economies of scales. Under such conditions, group selection should favor institutions and technologies that reduce this individual-group tension because such technologies or institutions allow populations to reap the benefits of economies of scale, or rather, economic growth [21]. One technology that might be favored is farming; however, group selection might also favor elaborate property rights by hunter-gatherers and the low-level food production of wild resources, or elaborate institutions of redistribution that “mask” emerging conflicts of interest in larger groups, depending on the local ecosystem. In any case, once a cultural adaptive shift has occurred, groups will continue to respond to population growth by living in larger groups and exploiting ecosystems more effectively.

For example, consider the archaeological record of the western Great Basin. Eerkens [39] describes a shift in economic emphasis in this area from the more communal, bulk processing of tubers and roots to the more individual based processing of seeds in pots among foragers around 600 BP. This shift is coincident with population growth and increases in group size. Eerkens’ argument for this shift focuses on an individual’s return rates for processing tubers vs. processing seeds. He argues that population growth led to larger group sizes and excessive sharing demands, and, in turn, individuals shifted their emphasis to the production of seeds because this resource had a higher return rate for individuals, once sharing demands are factored into a cost-benefit analysis, than other, more communal resources. In this argument it is easier to privatize seeds, which blunts the demands of excessive free-riders and, thus, the costs of excessive sharing [39].

The work-around hypothesis is similar to Eerkens’ argument, but emphasizes group selection processes. The key issue is that of free-riding. Although free-riders gain a short-term benefit, today’s free-riders are also tomorrows debtors. By free-riding too much individuals provide a logic for unequal status based on another individual’s precedent over resources. Based on the sub-linear scaling of population and territory size noted in the ethnographic data, I suspect that increases in group size in the prehistoric western Great Basin should have lead to the more efficient production of tubers due to increasing returns to labor. But this may have also coincided with more free-riding, as well as debt claims on the part of people who had first access to patches of tubers. The work-around hypothesis would suggest that this free-rider-debtor dynamic would result in the rapid cycling of individuals through camps and rampant distrust. In this case, group selection would favor those camp groups where individuals focused on seeds, a resource easier for individuals to produce and control, reducing the mistrust generated by the free-rider-debtor dynamic, allowing residential groups to function better as a collective.

Farming provides the same advantage as the shift to small-seeds noted above, just with a much higher energy potential than wild seeds. Framed from the temporal perspective of the archaeological record, sometimes farming is adopted to reduce the immediate tension between self-interest and cooperation in larger residential groups, which reduces the need to develop time consuming institutions that keep large groups functioning. However, farming has a much higher carrying capacity than foraging; thus, if populations continue to grow, eventually residential groups of farmers will far outstrip the absolute group sizes of foragers. From an archaeological standpoint, then, in some cases farming may have been adopted by individuals to blunt emerging social complexity. However, in doing so, the new found farmers sowed the seeds of ever increasing social complexity over the long-term, under the population growth regime of the Holocene, because farmers can live in much larger populations than foragers.

## Conclusion

The intensification of land use is a process associated with the evolution of human societies. How and why intensification occurs and its consequences for social evolution are a matter of intense research. In this paper, I have combined a model of territory size from ecology with cultural evolutionary theory from anthropology to investigate the processes that determine the territory sizes of agricultural societies. I have also proposed a work-around hypothesis for the decision to farm and used this hypothesis to compare the territorial dynamics of forager and farmer societies. The main results are that, consistent with forager societies, the composition of the diet and ethno-linguistic population size determine the territory sizes of agricultural societies. Similarly, the scaling relationship between territory size and population size is sub-linear in agricultural and forager societies. These macroecological patterns are consistent with the work-around hypothesis for the decision to farm.

I would like to end by mentioning two important directions for future research. First, we need to compare alternative models that may explain the sub-linear scaling of population and territory size across subsistence types in the first place. This scaling relationship may be the result of a universal law of energy use predicted by a metabolic theory of ecology [17]; alternatively, this scaling relationship might only result under specific technological or social circumstance suggested by social and economic theory [16]; or the sub-linear scaling relationship could be the result of bias in our statistical methods. Second, although the work-around hypothesis presented here is plausible, it needs to be tested against alternative hypotheses. For instance, one alternative is that individuals adopt farming simply because farming allows populations to live in larger groups and individuals recognize that by living larger groups they can generate further economies of scale. In economic parlance, economies of scale create a rising tide that lifts all boats, absent any “market imperfections.”

## Supporting Information

S1 Text Supplemental analysis. (PDF)

S1 Table Data on agricultural societies. (XLS)

S2 Text Ethnographic references for the agricultural societies in S2 Table. (ODT)

S2 Table Data on hunter-gatherer societies. (XLSX)
