# Supplementary material for: The Socioecology of Territory Size and a "Work-Around" Hypothesis for the Adoption of Farming
Source: PLoS One. 2016 Jul 8;11(7):e0158743. doi: 10.1371/journal.pone.0158743 (PMC4938390; doi:10.1371/journal.pone.0158743)
Supplement: S1 Text — (PDF) [file pone.0158743.s001.pdf]

The Socioecology of Territory Size: A comparison farmers and human foragers

Jacob Freeman*[1],

1 Anthropology Program, Utah State University, Logan, UT 84322.

Supporting Information

Table 1. All regression models run for the explanatory variables on the territory size of agricultural societies.

	(Intercept)	AE	Foraging	log(Population)	R^2	df	logLik	AIC	delta	weight
7	-1.46		0.07	0.81	0.66	4	-117.5	243.0	0.0	0.72
8	-1.79	0.00	0.07	0.82	0.66	5	-117.4	244.9	1.9	0.28
5	0.94			0.72	0.38	3	-135.5	277.1	34.1	0.00
6	1.72	-0.00		0.70	0.39	4	-135.3	278.6	35.6	0.00
4	7.43	-0.00	0.06		0.20	4	-143.2	294.5	51.5	0.00
3	6.00		0.06		0.18	3	-144.3	294.5	51.5	0.00
2	9.05	-0.00			0.05	3	-148.7	303.4	60.4	0.00
1	7.24				0.00	2	-150.2	304.5	61.5	0.00

Table 2. All regression models run for the explanatory variables on the territory size off forager societies.

	(Intercept)	HUNTING	log(CRR)	log(TLPOP)	MCM	R^2	df	logLik	AIC	delta	weight
16	2.23	0.03	-0.54	0.70	-0.02	0.65	6	-498.9	1009.7	0.0	1.00
8	2.65	0.04	-0.69	0.75		0.64	5	-507.4	1024.8	15.1	0.00
14	-0.96	0.04		0.66	-0.04	0.61	5	-520.8	1051.6	41.9	0.00
15	4.58		-0.67	0.64	-0.04	0.57	5	-537.5	1085.0	75.3	0.00
6	-2.06	0.05		0.74		0.54	4	-548.7	1105.4	95.7	0.00
7	6.20		-1.03	0.72		0.50	4	-561.4	1130.7	121.0	0.00
13	0.84			0.58	-0.06	0.49	4	-565.5	1139.1	129.4	0.00
12	6.20	0.02	-0.38		-0.04	0.43	5	-583.8	1177.6	167.9	0.00
10	3.74	0.03			-0.05	0.41	4	-590.7	1189.4	179.7	0.00
11	7.82		-0.50		-0.05	0.37	4	-599.7	1207.3	197.6	0.00
4	7.58	0.03	-0.66			0.36	4	-602.5	1213.0	203.3	0.00
9	4.72				-0.07	0.33	3	-611.1	1228.1	218.4	0.00
2	3.00	0.05				0.27	3	-625.1	1256.3	246.6	0.00
3	10.72		-0.98			0.25	3	-631.3	1268.5	258.8	0.00
5	-0.04			0.68		0.23	3	-635.5	1277.0	267.3	0.00
1	4.55					0.00	2	-679.1	1362.3	352.6	0.00

Note below is a spatial lag model meant to account for spatial autocorrelation among agricultural societies. Note that the population coefficient is statistically indistinguishable from the OLS models presented above.

```
SUMMARY OF OUTPUT: SPATIAL LAG MODEL - MAXIMUM LIKELIHOOD ESTIMATION
FOR THE AGRICULTURAL SOCIETIES
Data set             : AGPOP
Spatial Weight       : AGPOP2.gwt
Dependent Variable   :      LNArea  Number of Observations:   61
Mean dependent var   :     2.62295  Number of Variables   :    4
S.D. dependent var   :     1.21687  Degrees of Freedom    :   57
Lag coeff.   (Rho)   :    0.934426

R-squared            :    0.591494  Log likelihood        :     -69.571
Sq. Correlation      : -            Akaike info criterion :     147.142
Sigma-square         :    0.604909  Schwarz criterion     :     155.585
S.E of regression    :    0.777759

-----------------------------------------------------------------------
    Variable    Coefficient     Std.Error       z-value    Probability
-----------------------------------------------------------------------
    W_LNArea     0.9344262      0.0467022       20.00818     0.00000
    CONSTANT     -3.446972      0.4227135      -8.154394     0.00000
       LNPop     0.7788503     0.09480808        8.21502     0.00000
      FORAGE    0.03100545    0.004936569       6.280768     0.00000
-----------------------------------------------------------------------

REGRESSION DIAGNOSTICS
DIAGNOSTICS FOR HETEROSKEDASTICITY
RANDOM COEFFICIENTS
TEST                                        DF      VALUE        PROB
Breusch-Pagan test                          2         7.7154     0.02112

DIAGNOSTICS FOR SPATIAL DEPENDENCE
SPATIAL LAG DEPENDENCE FOR WEIGHT MATRIX : AGPOP2.gwt
TEST                                        DF      VALUE        PROB
Likelihood Ratio Test                       1         1.4199     0.23342

COEFFICIENTS VARIANCE MATRIX
   CONSTANT        LNPop       FORAGE    W_LNArea
   0.178687    -0.035880    -0.000794   -0.005850
  -0.035880     0.008989     0.000074    0.000028
  -0.000794     0.000074     0.000024    0.000001
  -0.005850     0.000028     0.000001    0.002181


  OBS          LNArea         PREDICTED         RESIDUAL         PRED ERROR
    1               4           3.46688          0.54142            0.53312
    2               2           3.40160         -1.42343           -1.40160
    3               4           4.34607         -0.35146           -0.34607
    4               3           3.32704         -0.33214           -0.32704
    5               4           3.99065          0.00950            0.00935
```

6	3	2.73027	0.27393	0.26973
7	4	3.98299	0.01728	0.01701
8	3	3.52407	-0.53223	-0.52407
9	4	3.42274	0.58625	0.57726
10	4	3.34842	0.66173	0.65158
11	3	3.25260	-0.25654	-0.25260
12	2	2.53104	-0.53931	-0.53104
13	4	3.40072	0.60861	0.59928
14	4	3.56746	0.43928	0.43254
15	3	3.70414	-0.71510	-0.70414
16	3	3.33493	-0.34014	-0.33493
17	0	1.20767	-1.22647	-1.20767
18	3	2.67771	0.32731	0.32229
19	2	1.54293	0.46419	0.45707
20	3	2.84088	0.16160	0.15912
21	3	3.24794	-0.25180	-0.24794
22	1	0.95473	0.04598	0.04527
23	3	3.25653	-0.26052	-0.25653
24	3	3.03228	-0.03278	-0.03228
25	3	3.19511	-0.19814	-0.19511
26	2	2.88992	-0.90378	-0.88992
27	4	3.45438	0.55412	0.54562
28	1	1.94322	-0.95791	-0.94322
29	2	2.07942	-0.08066	-0.07942
30	3	1.60444	1.41730	1.39556
31	4	3.57958	0.42697	0.42042
32	3	3.48740	-0.49499	-0.48740
33	4	3.68702	0.31785	0.31298
34	3	3.08612	-0.08747	-0.08612
35	1	1.23380	-0.23744	-0.23380
36	2	3.09433	-1.11137	-1.09433
37	4	2.30174	1.72470	1.69826
38	0	1.43375	-1.45608	-1.43375
39	4	3.02540	0.98978	0.97460
40	4	4.32835	-0.33347	-0.32835
41	3	1.75300	1.26642	1.24700
42	2	1.63441	0.37128	0.36559
43	1	1.01538	-0.01562	-0.01538
44	0	1.04356	-1.05981	-1.04356
45	4	2.36680	1.65864	1.63320
46	0	1.07499	-1.09173	-1.07499
47	2	2.56056	-0.56929	-0.56056
48	3	2.87807	0.12382	0.12193
49	3	2.08075	0.93356	0.91925
50	3	2.56336	0.44344	0.43664
51	3	2.54815	0.45889	0.45185
52	2	2.43240	-0.43914	-0.43240
53	3	2.34228	0.66796	0.65772
54	1	0.85922	0.14297	0.14078
55	3	3.52543	-0.53361	-0.52543
56	3	2.01380	1.00156	0.98620
57	3	3.30607	-0.31084	-0.30607

```
   58                0         1.56480        -1.58917        -1.56480
   59                0         1.22471        -1.24378        -1.22471
   60                3         1.47811         1.54559         1.52189
   61                2         1.21787         0.79431         0.78213
========================== END OF REPORT ==============================
```
