# Supplementary material for: The Socioecology of Territory Size and a "Work-Around" Hypothesis for the Adoption of Farming
Source: PLoS One. 2016 Jul 8;11(7):e0158743. doi: 10.1371/journal.pone.0158743 (PMC4938390; doi:10.1371/journal.pone.0158743)
Supplement: Supplementary file 2 [file pone.0158743.s003.odt]

Ethnographic and Environmental Data References:

Mapuche Huacho:

Faron, L. C., 1961. Mapuche Social Structure. University of Illinois Press, Urbana, IL.

Xhosa Shixini:

McAllister, P., 2001. Building the Homestead: Agriculture, Labour, and Beer in South Africa's Transkei. Ashgate Publishing Limited, Hamshire, UK.

Bhaca:

Hammond-Tooke, W.D. 1962. Bhaca Society: A people of the Transkeian uplands, South Africa. Oxford University Press, London.

Swazi:

Beemer, H. 1939. The diet of the Swazi in the protectorate. Bantu Studies 13: 200-236

Tswana (Tolkwa):

Gritvette, L. E. 1979. Kalahari agro‐pastoral‐hunter‐gatherers: The Tswana example. Ecology of Food and Nutrition 7:235-256.

Grtivette, L. E., 1978. Nutritional success in a semi-arid land: examination of Tswana agro-pastoralists of the Eastern Kalahari, Botswana. American Journal of Clinical Nutrition, 31, 1204-1220.

Cayua:

Watson, J. B. 1945., Historic influences and change in the economy of a southern Mato Grosso tribe. Acta Americana 3: 3-25

Pedi:

Monnig, H., O., 1967. The Pedi. J. L. Van Schaik Limited, Pretoria.

Betsileo:

Kottak, Conrad, P. 1980. The Past in the Present: History, ecology, and cultural variation in Highland Madagascar.  University of Michigan Press, Ann Arbor.

Gwembe Tonga:

Colson, E., 1960. Social Organization of the Gwembe Tonga. Manchester University Press, Manchester, UK.

Goba:

Lancaster, Chet S. 1981. The Goba of the Zambezi:Sex roles, economics and change. University of Oklahoma Press, Norman, OK.

Siriono:

Holmberg, H. R., 1969. Nomads of the Long Bow: The Siriono of Eastern Bolivia. The Natural History Press, Garden City, NY.

Lozi:

Gluckman, M., 1941. The Economy of the Central Bartose Plain. Rhodes-Livingston Institute, Livingston Northern Rhodesia.

Tikopia:

Firth, Raymond William. 1939. Primitive Polynesian economy. George Routledge & Sons, Ltd., London.

Matsigenka:

Johnson, A., 2003. Families of the Forest: The Matsigenka Indians of The Peruvian Amazon. University of California Press, Berkeley, CA.

Bemba:

Richards, A. I., 1939. Land, Labor and Diet in Northern Rhodesia: An Economic Study of the Bemba Tribe. Oxford University Press, London.

Wonie:

Otsuka, Ryutaro. 1983. Oriomo Papuans:Ecology of sago-eaters in lowland Papua. University of Totkyo Press, Tokyo.

Uchucmarca:

Brush, S. B., 1977. Mountain, Field, and Family: The Economy and Human Ecology of an Andean Village. University of Pennsylvania Press, Pittsburg, PA.

Maring (Bomagai-Angoiang):

Clarke, William C. 1971. Place and People: An ecology of a New Guinean Community. University of California Press, Berkeley, CA.

Tsembaga:

Rappaport, R. A., 1968. Pigs for the Ancestors: Ritual in the Ecology of a New Guinea People. Yale University Press, New Haven, CT.

Sandawe:

Newman, J. L., 1970. The Ecological Basis of Subsistence Change among the Sandawe of Tanzania. National Academy of Sciences, Washington DC.

Nuaulu:

Ellen, R. F., 1978. Nuaulu Settlement and Ecology. Verhandelingen, The Hague.

Ka`apor:

Balee, William, 1994. Footprints of the Forest: Ka'apor Ethnobotany-the Historical Ecology of Plant Utilization by an Amazonian People. Columbia University Press, New York.

Tana Torja:

Nooy-Palm, Hetty. 1979. The Sa'dan-Toraja: a study of their social life and religion V. 1.  Martinus Nijhoff, Hague.

Sonjo:

Gray,Robert F. 1963. The Sonjo of Tanganyka: An anthropological study of an irrigation based society. Greenwood Press, Westport, CT.

Boyela (4 subtribes):

Sato, Hiroaki 1983. Hunting of the Boyela, Slash-and-Burn Agriculturalists, in the Central Zaire Forest. African Study Monographs 4: 1-54.

Amba:

Winter, E.H. 1955. Bwamba Economy: The development of a primitive subsistence economy in Uganda. East African Institute of Social Research, Kampala.

Sebei (Sasur):

Goldschmidt, W., 1976. Culture and Behavior of the Sebei. University of California Press, Berkeley, CA.

Yanoama:

Smole, W. J., 1976. The Yanoama Indians: A Cultural Geography. University of Texas Press, Austin, TX.

Iban:

Freeman, D., 1970. Report on the Iban. Humanities Inc. Press, New York. &

Nukak:

Politis, G. G., 2007. Nukak: ethnoarchaeology of an Amazonian people. Left Coast Press, Walnut Creek, CA.

Lugbara:

Middleton, J. and Greenland, D. J., 1954. Land and population in West Nile District, Ugnada. The Geographical Journal, 120, 446-455.

Semai:

Dentan, R. K., 1968. The Semai: A Nonviolent People of Malaya. Holt, Rinehart and Winston, New York. & Dentan, R. K., 1971. Some Senoi Semai planting techniques. Economic Botany, 25, 136-159.

Guahibo:

Morey, R. V. and Metzger, D. J., 1974. The Guahibo: people of the savanna. Engelbert, Stiglmayr, Los Angeles.

Azande:

De Schlippe, P., 1956. Shifting Cultivation in Africa: The Zande System of Agriculture. Routledge and Keagan Paul, London.

Baxter, P.T.W. and Butt, Audrey. 1953. The Azande, and related peoples of the Anglo-Egyptian Sudan and Belgian Congo. Ethnographic survey of Africa, East Central Africa, part 9. Daryll Forde ed. International African Institute, London.

NE Igbo:

Jones, Gwilym Iwan. 1961. Ecology and social structure among the North Eastern Ibo. Africa 31:117-134.

Panare:

Henley, Paul, 1982. The Panare: Tradition and change on the Amazonian frontier. Yale University Press, New Haven.

Tiv:

Bohannan, L. and Bohannan, P., 1953. The Tiv of Central Nigeria. International African Institute, London.

Nuer:

Evans-Pritchard, E. E., 1947. The Nuer: A Description of the Modes of Livelihood and Political Institutions of a Nilotic People. The Clarendon Press, Oxford, UK.

Kofyar:

Netting, R. McC., 1968. Hill farmers of Nigeria: Cultural ecology of the Kofyar of the Jos Plateau. University of Washington Press, Seattle, WA.

Ulithi:

Lessa, William Armand 1950. The ethnography of Ulithi Atoll. University of California, Los Angeles.

Yukpa:

Ruddlle, Kenneth 1974. The Yukpa cultivation system: A study of shifting cultivation in Colombia and Venezuela. University of California Press, Berkeley, CA.

Hanunoo:

Concklin, H. C., 1957. Hanunoo Agriculture. Food and Agriculture Organization of the United Nations, Rome, Italy.

Miskito:

Nietschmann, B., 1973. Between Land and Water: The Subsistence Ecology of the Miskito Indians, Eastern Nicaragua. Seminar Press, New York.

Dogon:

Monserrat, P. M., 1957. The Dogon. Universitaires de France, Paris.

Eritrea Highland:

Tronvoll, K., 1998. Mai Weini: a highland Village in Eritrea. The Red Sea Press, Lawrenceville, NJ.

Maya (Kekchi):

Wilk, R. R., 1997. Household Ecology: Economic Change and Domestic Life among the Kekchi Maya in Belize. Northern Illinois University Press, Dekalb, IL.

Ifugao (subarea):

Conklin, Harold C 1967. Some aspects of ethnographic research in Ifugao. Transactions of the New York Academy of Sciences, Ser. II. 30:99-121.

Lamet:

Izikowitz, Karl Gustav. 1951. Lamet: Hill peasants in French Indochina. Ethnologiska Museet, .

Khasi:

McCormack, A., 1964. Khasis. Ethnic groups of mainland Southeast Asia (M. F. Lebar, G. C. Hickey, J. K. Musgrave), pp. 105-112. Human Relations Area Files, New Haven, CT.

Garo:

Majumdar, D. N., 1978. A Study of culture change in two Garo villages. Anthropological Survey of India, Govt. of India, Calcutta, India.

Playfair, A., 1909. The Garos. David Nutt, London.

Tarahumara:

Kennedy, J., 1978. Tarahumara of the Sierra Madre: Beer, Ecology, and Social Organization. AHM Publishing, Arlington Heights, IL.

Hasinai:

Griffith, W. J., 1954. The Hasinai indians of East Texas as seen by Europeans. Middle American Research Institute, Tulane University, 11, 41-168.

Cocopa:

Kelly, W., 1977. Cocopa Ethnography. University of Arizona Press, Tucson, AZ.

Creek:

Swanton, J. R., 1922. Early History of the Creek Indians and Their Neighbors. Smithsonian Institution, Bureau of American Ethnology, Washington DC.

Mohave:

Stewart, Kenneth M. 1983. Mohave. In Handbook of North American Indians: Southwest V. 10. A. Ortize ed. pp. 55-70. Smithsonian Institution Press, Washington DC.

Castetter, E. F. and Bell, H. W., 1951. Yuman Indian Agriculture; Primitive Subsistence on the Lower Colorado and Gila Rivers. University of New Mexico Press, Albuquerque, NM.

Havasupai:

Whiting, A. F., 1985. Havasupai Habitat. University of Arizona Press, Tucson, AZ.

Spier, L., 1928. Havasupai Ethnogrphy. American Museum of Natural History, New York.

Hunzakut:

Sidky, M. H. 1993.  Subsistence, ecology, and social organisation among the Hunzakut: A high-mountain people in the Karakorams. Eastern Anthropologist 46:145-170.

Powhatan:

Binford, L. R., 1991. Cultural Diversity among Aboriginal Cultures of Coastal Virginia and North Carolina. Garland Publishing Inc., New York.

Delaware:

Goddard, I., 1978. Delaware. In the Handbook of North American Indians (ed.) Sturtevant, W., C. Smithsonian Institution, Washington D. C.

Newcomb, W. W., 1956. The Culture and Acculturation of the Delaware Indians. University of Michigan Press, Ann Arbor, MI.

Huron:

Trigger, B., 1969. The Huron. Holt, Rinehart and Winston, Inc., Fort Worth, TX.

Hidatsa:

Stewart, Frank Henderson. 2001. Hidatsa. In Handbook of North American Indians, Vol. 13: Plains. Demallie, R. J. ed. Smithsonian Institution Press, Washington, DC.

FAO Annual Actual Evapotranspiration Map:

http://ref.data.fao.org/map?entryId=750d56ca-f92a-405e-8bbc-302221d55917&tab=about
